# Encapsulation of bioactive compounds extracted from date palm seeds (*Phoenix dactylifera* L.) and their use in functional food

**DOI:** 10.3389/fnut.2022.1051050

**Published:** 2022-11-23

**Authors:** Mahmood A. Hashim, Xin Huang, Liudmila A. Nadtochii, Denis A. Baranenko, Mohamed Said Boulkrane, Tamer M. El-Messery

**Affiliations:** ^1^International Research Center “Biotechnologies of the Third Millennium”, Faculty of Biotechnologies, ITMO University, Saint Petersburg, Russia; ^2^Agricultural Research Centre, Food Technology Research Institute, Giza, Egypt; ^3^Department of Food and Nutrition, Faculty of Agriculture and Forestry, University of Helsinki, Helsinki, Finland; ^4^Faculty of Ecotechnologies, ITMO University, Saint Petersburg, Russia; ^5^Dairy Department, National Research Centre, Cairo, Egypt

**Keywords:** bioaccessibility, bioactive substances, liposome, functional yogurt, encapsulation

## Abstract

Liposomes have been used as a novel phytoconstituent delivery system to encapsulate lyophilized palm seed phenolic extract (PSPE) and incorporate it into yogurt as a food model to enhance the bioavailability of PSPE. Phenolic compounds were extracted with aqueous ethanol from palm seed powder using the solvent-maceration approach assisted by ultrasonication. Lyophilized PSPE (0.2–1% *w/v*) was enclosed in a liposome structure coated with or without chitosan (primary/secondary liposome). Particle size, zeta potential, encapsulation efficiency (EE), Fourier transform infrared spectroscopy (FTIR), and transmission electron microscopy (TEM) were applied to investigate the primary and secondary liposomes. To assess the *in vitro* bioaccessibility of PSPE and primary/secondary liposomes, the total phenolic content (TPC) and the antioxidant activity were studied during the oral, gastric, and intestinal digestion stages. Three concentrations of lyophilized secondary liposomes (1.25, 2.5, and 3.75% *w/v*) were added to the yogurt food model. During the 14 days of storage, the physical, chemical, and sensory properties were assessed. Compared to the primary liposomes (87%), the secondary liposomes (91%) showed a higher encapsulation efficiency. Comparing the secondary liposomes to the original liposomes and the non-encapsulated PSPE, the bioaccessibility of phenolic compounds was improved. Fortified yogurt with secondary liposomes had a lower syneresis and viscosity than the reference yogurt. The encapsulated PSPE provided a good level of protection, and its release increased throughout the intestinal phase. Thus, PSPE in a microencapsulated form has been proven to be a rich and cost-effective source of phenolics that can be used successfully to produce functional yogurt.

## Introduction

Yogurt is the most common fermented milk product. Its popularity is attributed to its functional properties (1). Therefore, yogurt represents an integral part of the human diet. Although yogurt is an excellent source of macronutrients and has many bioactive ingredients, it is not a rich source of polyphenols (2).

Fruit and vegetables are essential sources of bioactive substances, including polyphenols; however, they experience significant losses (from 20% to approximately 50%) throughout the production process. Thus, it becomes challenging to recover and recycle food waste for edible uses (3).

Date palm, *Phoenix dactylifera* L., is a crucial fruit-bearing tree grown widely in the Middle East and is a staple sustenance for millions worldwide. The production of dates increased by a million metric tons between 2010 and 2018. Approximately 8.53 million metric tons of dates were produced worldwide in 2018, up from 7.53 million metric tons in 2010. According to an FAO report (4), Egypt ranked the highest in the world among date-producing countries, with the production volume amounting to almost 1.7 million metric tons of dates that year. Palm seed comprises approximately 10–15% of the weight of the date fruit, thus producing a vast mass of bioresources ready for exploitation into a value-added product (5).

The most important part of the fruit for the plant to survive is the seed, which also contains a substantial proportion of metabolites that play a crucial role in interacting with free radicals and averting their harm when consumed. It is also associated with neurodegenerative and metabolic illnesses of plant damage (6). The human consumption of these compounds has been shown to provide several advantages, including antioxidant, anticarcinogenic, antibacterial, antimutagenic, and anti-inflammatory qualities that lower the risk of cardiovascular disease (7). Palm seeds are rich in phenolics and flavonoids. For example, their phenolic and antioxidant contents ranged from 3,102 to 4,430 mg of gallic acid equivalent per 100 g and 58,000 to 92,900 mmol of Trolox equivalent per 100 g, respectively (8).

Thus, palm seeds appear to be a prospective source of polyphenols for the human diet. On the other hand, their direct use presents certain issues. The primary drawbacks of palm seed polyphenol extract powder include its low-solubility aqueous phase, unpleasant taste, susceptibility to high temperature, destruction during food manufacturing, alkaline conditions, and storage. Additionally, intestinal enzymes hydrolyze polyphenols during digestion before absorption, thus reducing the accessibility of polyphenols. In addition, only 5–10% of polyphenols may be absorbed in the intestinal stage of digestion, with the remainder being eliminated in the feces after the buildup in the large intestine, which affects the accessibility of polyphenols. During absorption, all polyphenols primarily accumulate in the small intestine and the liver (7). It is not easy to successfully preserve those contained in food items with enough activity, necessitating the employment of an appropriate delivery system.

Encapsulation presents a solution to the above problems and the potential to improve the amount of polyphenols absorbed and their antioxidant activity in food products (9). Several encapsulation techniques have been employed to preserve various polyphenols, including spray drying (10), freeze-drying (11), nanoprecipitation (12), emulsions (13), liposomes (14), and phytosomes (15). However, using organic solvents and non-food-grade materials, complex and costly equipment, expensive encapsulating materials, reduced encapsulation efficiency, non-stable capsules, and large particle diameters all reduce the sensory attributes and performance of food products. Liposomes, as a new delivery vehicle in food products, do not seem to have received much attention (14), but they might provide a solution to the above problems.

Elegant liposome bilayer vesicles are an ideal encapsulation technology due to their biocompatibility, biodegradability, reduced particle size, and ability to transport a wide range of bioactive substances that can be incorporated within and enclosed by a phospholipid membrane. Soy lecithin is one of the phospholipids used in the liposome methodology; when phenolic compounds are integrated into these phospholipids, an innovative formulation known as “phenolipids” is formed, providing a novel use for encapsulated phenolics in the pharmaceutical and food industries (16).

This research aimed to preserve polyphenols derived from palm seed powder from the destruction caused by food manufacturing, storage, and digestion by encapsulating them inside soy lecithin liposomes. In addition to encapsulation efficiency, the bioaccessibility of encapsulated polyphenols *in vitro* was investigated for antioxidant activity and phenolic content. A set of yogurt was used as the liposome insertion vehicle to improve phenolic compound bioaccessibility with maximal antioxidant retention.

## Materials and methods

### Materials

Palm seed (PS) cultivars, namely, “Medjool, Amri, and Siwi”, were procured from Giza's Central Laboratory for Palm Research and Development. Soy lecithin nutritional supplement granules obtained from a non-genetically modified organism (GMO) soybean were acquired from Solgar company (Leonia, NJ 07605 USA). All enzymes and chemicals were acquired from Sigma-Aldrich Co. Yogurt starter culture (YC-X11) containing *Streptococcus thermophilus* and *Lactobacillus bulgaricus* was received from Chr. Hansen laboratory (Hoersholm, Denmark).

### Methods

#### Palm seed powder preparation

Palm seed powder was prepared from 10 kg of palm fruits harvested at the full ripeness stage from the date palm tree and granted by the Central Laboratory for Palm Research and Development (Giza, Egypt). The seeds were rinsed with distilled water to remove any remaining palm flesh before oven-drying for 48 h at 50°C. Palm seeds from each cultivar were ground in a heavy-duty cutting mill SM100 (Retsch, GmbH company, Haan, Germany) with a 1.5 kW drive and 1,500 rpm rotor speed and passed through a 1–2 mm sieve.

#### Preparation of phenolic extract from palm seed powder

A Branson digital Sonifier SFX 250 (Emerson Electric Co., Ferguson, USA) was used to sonicate 10 g of PSPE in 200 mL of ethanol (70% *v*/*v*) for 30 min at 250 watts of power at 20 k*Hz* to obtain PSPE extraction. The experiment included three identical extraction procedures. The latter was kept at room temperature surrounding the beaker with an ice bath to prevent overheating. The supernatant of the three extractions was combined after centrifuging at 12,000 × *g* for 30 min. The solvent was evaporated using a rotary evaporator (BÜCHI Labortechnik AG, Flawil, Switzerland). The residue was dried in a freeze dryer Gamma 2-16 LSC plus (Osterode am Harz, Germany) at −52°C for 48 h at 1.03 mbar and stored at −18°C. The freeze-dried phenolic powder was further dissolved in distilled water at a ratio of 1% (*w/v*) for analyses of total phenolic content and antioxidant activity.

#### Determination of the total phenolic content (TPC)

The total phenolic content was calculated in the PSPE using the Folin-Ciocalteu method described by Al-Farsi et al. (17). An aliquot of 0.6 mL of distilled water (DW) and 0.2 mL of Folin-phenol Ciocalteu's reagent (diluted from 10-fold stock solution with distilled water) were added to 0.2 mL of dissolved phenolic powder. Five minutes later, 3 mL of DW and 1 mL of saturated sodium carbonate solution (8% *w*/*v*) were added to the mixture. The mixture was incubated in the dark for 30 min, and after that, the absorbance was measured at a wavelength of 765 nm. The phenolic content was calculated as the proportion of gallic acid (mg) per gram of sample.

#### Determination of antioxidant activity

##### DPPH radical scavenging activity assay

DPPH (2,2-diphenyl-1-picryl-hydrazyl-hydrate) free radical assay was carried out according to the method of Boly et al. (18). Briefly, 100 μL of freshly prepared DPPH reagent (0.1% in methanol) was added to 100 μL of the sample in a 96-well plate (*n* = 3). The mixture was incubated at room temperature for 30 min in the dark. At the end of the incubation, the resulting reduction in DPPH color intensity was measured at 520 nm. The results are represented as the mean ± standard deviation (SD) according to the following equation:


(1) $$\begin{array}{l} \begin{array}{l} \begin{array}{l} \text{Inhibition of DPPH (\%) }\\ \text{=}\frac{\text{(Abs of control- Abs of the sample)}}{\text{Abs of control}}\text{ ×100} \end{array} \end{array} \end{array}$$

##### FRAP ferric reducing antioxidant power assay

This assay was conducted according to the method of Benzie and Strain (19), with minor modifications for experiments in microplates, with TPTZ (2,4,6-tripyridyl-s-triazine) reagent (300 mM acetate buffer (pH 3.6), 10 mM TPTZ in 40 mM HCl, and 20 mM FeCl_3_, with a ratio of 10:1:1 *v/v/v*). A total of 190 μL of freshly prepared TPTZ reagent was mixed with 10 μL of the extraction sample in a 96-well plate (*n* = 3), and the mixture was incubated at room temperature for 30 min in the dark. At the end of the incubation period, the resulting blue color was measured at 593 nm wavelength. Data are represented as the mean value ± SD.

Trolox TE (6-hydroxy-2,5,7,8-tetramethylchroman-2-carboxylic acid) was used as a reference in both methods, and a stock solution of 100 μM Trolox was prepared in methanol, from which 7 concentrations of 50, 40, 30, 20, 15, 10, and 5 mM were prepared. The activity of the samples is presented as the ratio of (mM TE) and (mg) samples using the linear regression equation extracted from the calibration curve (linear dose-response curve of Trolox).

#### Preparation of primary and secondary liposomes

Two grams of soy lecithin was dissolved in 100 mL of acetate buffer (pH 3.7, 0.1 M). The lyophilized PSPE (0.2, 0.4, 0.6, 0.8, and 1% *w/v*) was dissolved in soy lecithin solution. A high shear disperser (DI-25 Yellow line, IKA) was used for 10 min at 9,500 rpm to homogenize the lecithin dispersion. To reduce the size of the primary liposomes with or without PSPE, a sonicator (160 W power, 20 k*Hz* frequency, and 50% pulse, Sonics, Vibra, Cell, USA) was applied. To avoid sample heating, the homogenizer chamber was chilled with cold water throughout the homogenization process. The layer-by-layer deposition was used to create secondary liposomes. Chitosan (0.4% *w/v* dissolved in acetate buffer solution pH = 3.7, 0.1 M) was added to the primary liposome and stirred overnight at 200 rpm at room temperature. As a result, a positively charged chitosan coating was applied to the surface of negatively charged primary liposomes.

### Primary and secondary liposome descriptions

The ζ-potential and particle size of the liposomes were identified according to the method of González-Ortega et al. (20). First, a particle charge titration analyzer (Zetasizer Nano ZS from Malvern Instruments, Worcestershire, UK) was used to calculate the ζ potential. Next, a dynamic light scattering equipment was used to determine the particle size distribution (Mastersizer MS3000, Malvern Instruments, Worcestershire, UK). Finally, the mass median diameter (MMD) Dv50 was used to calculate the average particle size.

#### Encapsulation efficiency (EE)

The pellet containing liposomes was collected by centrifugation (12,000 × *g*, 180 min, 20°C) according to González-Ortega et al. (20), and the supernatant containing free phenolic compounds (non-encapsulated) was examined. To determine the amount of encapsulated phenolic compounds, 1 mL of methanol and 1 mL of chloroform (1:1*, v/v*) were used to disturb the resuspended pellets of liposomes. The mixture was vigorously vortexed before phase separation. The phenolic concentrations in the upper water-methanol phase and the supernatant were measured, and the encapsulated and non-encapsulated fractions were calculated using these values. To calculate the encapsulation efficiency, Equation (2) was applied:


(2) $$\begin{array}{l} \begin{array}{l} \begin{array}{l} \text{Encapsulation Efficiency}\;(\%)\\ =\frac{mass\;\;of\;TPC\;-mass\;\;of\;FPC\;}{TPC}\times 100 \end{array} \end{array} \end{array}$$

TPC: Total phenolic compounds (encapsulated + non-encapsulated)
FPC: Free phenolic compounds in the supernatant (non-encapsulated)

#### Fourier transform infrared spectroscopy (FT-IR)

Freeze-dried encapsulated phenolic samples were examined using attenuated total reflectance (ATR)-FTIR, Bruker VERTEX 80 (Germany), against a diamond crystal. The spectrum was collected in the range of 4,000–500 cm^−1^ at a resolution of 4 cm^−1^ and a refractive index of 2.4. The functional groups of the NH, CH_2_, PO_2_, C=O, and C-O-C groups were compared among primary and secondary liposomes, and non-encapsulated phenolic samples and lecithin were also measured as controls.

#### Transmission electron microscopy (TEM)

Twenty microliters of encapsulated samples were deposited on a film-coated 200-mesh copper specimen grid for 10 min, and the surplus fluid was collected using filter paper. The grid was then dyed with one drop of 3% (*w/v*) phosphotungstic acid and dried for 3 min. After drying, the coated grid was studied under a TEM microscope (JEM-2100 Electron Microscope, JEOL CO., Ltd., Beijing, P.R. China) at 160 kV to screen the samples.

#### *In vitro* digestion study (bioaccessibility)

Encapsulated and non-encapsulated lyophilized PSPE coated and not coated with chitosan were subjected to an *in vitro* digestion procedure simulating oral, gastric, and intestinal digestion as reported by El-Messery et al. (21). All samples were digested at 37°C with constant shaking at 50 rpm in the order of simulated salivary fluid (SSF), simulated gastric fluid (SGF), and simulated intestinal fluid (SIF). First, 1 ml of each sample was digested with 5 mL of SSF that included 7.5 mg of α-amylase (300 U/mg protein) and 25 μL of 0.3 M CaCl_2_. This solution was mixed, and the pH was adjusted to 7 by 0.1 M NaOH and incubated for 2 min. The pH of the oral digestate was adjusted to 3.0 by 0.1 M HCl, and gastric digestion was initiated by combining 5 mL of SGF, including 20 mg of pepsin (2,000 U/mL) and 0.15 mM CaCl_2_, and incubated for 2 h. The pH of the gastric digestate was adjusted to 7, and intestinal digestion was initiated by combining 10 mL of SIF containing 37.5 mg of pancreatin (100 U/mL), 0.6 mM CaCl_2_, and 40 mg of bile salts. The intestinal digestion was incubated for 2 h. Before analysis, all soluble fractions were centrifuged and filtered for the total phenolic content and antioxidant activity (DPPH and FRAP procedures) according to the methods of Al-Farsi et al. (17), Boly et al. (18), and Benzie and Strain (19). Undigested PSPE samples were also measured as controls.

### Yogurt preparation

Yogurt preparations were produced from fresh, low-fat cow milk with 0.5% fat, 3.7% protein, and 4.9% lactose that was pasteurized at 95°C/10 min and then cooled to 42°C. The milk samples were inoculated with a yogurt culture (*S. thermophilus* and *lb. delburkii* ssp. *bulgaricus*) at 3% (*w/v*) and incubated at 42°C for 3–4 h until the pH dropped to 4.6.

The yogurt was divided into four portions: the first portion representing control yogurt (labeled C) was prepared without any additives, and the other portions, T1, T2, and T3, were yogurts containing 1.25, 2.5, and 3.75 g lyophilized secondary liposomes equivalent to 25, 50, and 75 mg of phenolic compounds, respectively. All yogurt samples were stored at 4°C for 15 days. This experiment was replicated three times.

### Yogurt's physical and chemical properties

#### Acidity and pH

The pH of the yogurts was measured immediately after manufacture at 25°C using a pH meter (pH 211, HANNA Instruments, Leighton Buzzard, UK). The titration method was used to determine the acidity as lactic acid (22).

#### Syneresis of yogurt

According to the method of El-Messery et al. (23), the syneresis rate of yogurt was calculated using the following Equation (3):


(3) $$\begin{array}{l} \text{Syneresis }\left(\text{\%}\right)=\frac{weight\;of\;the\;supernatant}{\;weight\;of\;yogurt\;sample}\;X\;100 \end{array}$$

#### Color

Hunter LAB (Color quest XE, Stotto Hunter Lab, Leicester LE4 3EH, UK) was used to evaluate the color parameters of the yogurt samples. A D65 illuminant was used as the light source, and the viewing angle was 10 degrees. The color was evaluated using L^*^ (lightness), a^*^ (the negative value indicates green, the positive value indicates red), and b^*^ (the negative value means blue, and the positive value means yellow).

#### Texture profile examination (TPA)

The texture profile analysis (TPA) of the yogurt samples was conducted using the two-fold compression test (Multi test 1d Memesin, Food Technology Corporation, Slinfold, W. Sussex, UK). Compression tests were conducted at room temperature to plot force (N) vs. time (s). A 25 mm diameter perplex conical-shaped probe was used to examine the samples at five locations on their surface. The samples were first compressed by 30% of their initial depth at a rate of 2 cm/min throughout the pretest, compression itself, and relaxation of the sample. Using the International Dairy Federation's definition, the following variables were extracted from the force-time curve (IDF, 1991). The maximum first compression force (N) refers to hardness. The area under the second compression divided by the area under the first compression (A2/A1) is used to measure cohesiveness. Adhesiveness (N.s) is the negative area of the curve (A3). Springiness (mm) refers to the ratio of the second compression length to the first compression length (L2/L1). Gumminess (N) = hardness × cohesiveness; Chewiness (Jm) g/mm = gumminess × springiness.

#### Sensory evaluation

The evaluation process was performed when fresh and after 7 and 15 days of yogurt storage using a form prepared according to a 10-point hedonic scale that was selected for sensory evaluation. In terms of sensory properties, the appearance, flavor, and texture were assessed using a form according to yogurt standards. Each yogurt sample was labeled and randomly given to panelists in separate three-digit-coded plastic cups. The sensory characteristics were repeated three times.

#### Statistical analyses

The mean values and the standard deviation (SD) for each yogurt treatment were calculated after the three trials. The data were analyzed with SPSS (version 16.0), and Duncan's test was conducted with an α significance level of 5%.

## Results and discussion

### Primary and secondary liposome descriptions

Table 1 shows the characteristics of primary and secondary liposomes based on particle size, ζ-potential, and encapsulation efficiency. The results suggest that an increase in PSPE addition into liposomal dispersions increased the particle size. This increase may be due to the creation of cross-links among phospholipids and polyphenolic compounds. The lipophilicity of PSPE prompted its insertion on the liposome surface and might cause hydrogen bonding between polar head groups and the phenolic compounds in the extract (10). As a result, more PSPE might react with the lipid structure of liposomes, potentially leading to the production of the largest particles in liposomes. Adding chitosan (cationic polymer) to the primary liposome induced a two- to three-fold increase in liposome particle size. The particle size was also increased by increasing the PSPE concentration. These findings are similar to those of subsequent studies. Ramli et al. (24) also found that by increasing the concentration of encapsulated stingless bee extract, the liposome particle size was significantly increased similarly (25), and the mean particle size of the green tea extract-loaded liposomes was higher than the unloaded liposome mean particle size.

**Table 1:** Particle size, ζ-potential, and encapsulation efficiency of primary and secondary liposomes.

**PSPE conc. in liposome (%)**	**Samples size (nm)**		ζ **potential (mV)**		**Encapsulation efficiency (%)**	
	**Primary liposomes**	**Secondary liposomes**	**Primary liposomes**	**Secondary liposomes**	**Primary liposomes**	**Secondary liposomes**
0	99.00 ± 0.02^f^	90.00 ± 0.04^f^	−13.2 ± 0.4^f^	20.8 ± 0.2^f^	ND	ND
0.2	101.00 ± 0.05^e^	177.00 ± 0.03^e^	−11.9 ± 0.2^e^	18.9 ± 0.4^e^	71.0 ± 0.8^d^	85.7 ± 0.9^c^
0.4	111.00 ± 0.03^d^	158.00 ± 0.02^d^	−11.1 ± 0.3^d^	19.8 ± 0.8^d^	81.6 ± 0.4^c^	91.0 ± 0.4^b^
0.6	131.00 ± 0.05^c^	171.00 ± 0.02^c^	−9.8 ± 0.4^c^	17.7 ± 0.6^c^	81.8 ± 0.9^c^	90.7 ± 0.5^b^
0.8	138.00 ± 0.08^b^	883.00 ± 0.05^b^	−10.4 ± 0.4^b^	14.1 ± 1.1^b^	84.9 ± 0.3^b^	91.5 ± 0.4^a^
1	158.00 ± 0.03^a^	1350.00 ± 0.03^a^	−9.0 ± 0.2^a^	13.2 ± 0.9^a^	86.7 ± 0.7^a^	91.4 ± 0.8^a^

The data represent the average value ± standard deviation of three replicates from each sample. Different letters in the columns represent statistically significant differences (*P* < 0.05). ND, not determined.

Furthermore, chitosan-coated primary liposomes and the ζ-potential shifted from the negative charge into a positive charge, as shown in Table 1. Therefore, the increase in particle size and the change in liposome surface charge are two essential factors for efficient surface coating. Previous research has shown that adding negative charges to liposomal anionic diffusion improves the negativity of the overall ζ-potential (26).

The encapsulation efficiency of the primary and secondary liposomes is presented in Table 1. The EE of encapsulated phenolics in liposomes has been evaluated in several studies (26, 27). Previous studies (28, 29) have shown that phenolic concentration may improve the liposome's EE. According to our results, the EE of primary and secondary liposomes gradually increased with increasing PSPE concentrations. On the other hand, secondary liposomes exhibited significantly higher EE than primary liposomes at all PSPE concentrations. Hence, we used secondary liposomes for yogurt integration. Indeed, the EE findings are similar to those obtained in an earlier study by Akgün et al. (30), where the encapsulation efficiency of primary and secondary liposomes with 0.1% (*w/w*) blackberry waste extract was 71.2 and 78.5%, respectively.

#### *In vitro* digestion study (bioaccessibility)

The bioaccessibility of PSPE primary and secondary liposomes was determined using the *in vitro* gastrointestinal digestion model illustrated in Table 2. The findings revealed that the non-encapsulated PSPE had the lowest bioaccessibility (the less bioactive compounds detected during analysis and the low amount of bioactive compounds that are available for absorption in the gut after digestion) compared with both primary and secondary liposomes (the bioactive compounds were protected against digestion, and more bioactive compounds were available for absorption). This result might be due to the hydrophobic nature of polyphenols, which reduce water solubility. The same findings were observed in an *in vitro* model and found that the intestinal bioaccessibility of the non-encapsulated tea polyphenol-based nutraceutical formulation was significantly low (ranging from 13.00 to 23.77) (31).

**Table 2:** Changes in the total phenolic content (TPC) and antioxidant activities (DPPH and FRAP) of PSPE powder, primary and secondary liposomes, and *in vitro* digestion.

**Treatment**	**Conc. (%)**	**TPC eq (Gallic acid** μ**g/mL)**			**DPPH eq mM Trolox**			**FRAP eq mM Trolox**		
		**Oral**	**Gastric**	**Intestinal**	**Oral**	**Gastric**	**Intestinal**	**Oral**	**Gastric**	**Intestinal**
Primary liposomes	0.2	2.7 ± 0.2^cd^	2.4 ± 0.8^e^	27.1 ± 7.1^a^	0.01 ± 0.03^f^	1.29 ± 0.10^c^	1.59 ± 0.03^d^	0.26 ± 0.04^b^	0.38 ± 0.02^bc^	0.50 ± 0.03^d^
	0.4	4.05 ± 0.11^b^	5.3 ± 0.5^c^	25.1 ± 4.0^ab^	0.39 ± 0.02^cd^	1.94 ± 0.22^a^	2.39 ± 0.01^a^	0.27 ± 0.03^b^	0.36 ± 0.01^c^	0.60 ± 0.01^c^
	0.6	2.3 ± 2.9^d^	13.1 ± 2.3^a^	22.2 ± 5.8^b^	1.40 ± 0.04^a^	1.48 ± 0.12^b^	1.82 ± 0.41^c^	0.42 ± 0.05^a^	0.52 ± 0.07^a^	0.77 ± 0.11^b^
	0.8	3.1 ± 0.3^c^	9.2 ± 0.2^b^	27.3 ± 7.2^a^	1.49 ± 0.06^a^	1.48 ± 0.24^b^	1.82 ± 0.13^c^	0.39 ± 0.06^ab^	0.47 ± 0.04^ab^	0.84 ± 0.08^a^
	1.0	5.1 ± 2.6^a^	6.3 ± 2.7^c^	28.5 ± 7.5^a^	1.11 ± 0.13^ab^	1.95 ± 0.32^a^	2.39 ± 0.11^a^	0.35 ± 0.09^c^	0.38 ± 0.06^bc^	0.70 ± 0.08^b^
Secondary liposomes	0.2	1.9 ± 0.2^e^	2.30 ± 0.01^e^	10.2 ± 2.3^c^	0.18 ± 0.05^d^	0.41 ± 0.02^f^	0.84 ± 0.01^f^	0.22 ± 0.05^b^	0.27 ± 0.02^de^	0.28 ± 0.01^f^
	0.4	2.39 ± 0.14^d^	5.86 ± 0.02^c^	9.5 ± 2.7^c^	0.59 ± 0.04^c^	0.85 ± 0.01^de^	1.12 ± 0.11^e^	0.25 ± 0.02^ab^	0.30 ± 0.01^d^	0.36 ± 0.01^ef^
	0.6	2.13 ± 0.11^d^	9.9 ± 2.5^b^	12.0 ± 2.4^c^	0.07 ± 0.10^f^	0.77 ± 0.01^e^	1.75 ± 0.21^c^	0.22 ± 0.01^ab^	0.39 ± 0.01^bc^	0.39 ± 0.01^ef^
	0.8	1.89 ± 1.6^e^	4.6 ± 2.5^d^	13.2 ± 3.1^c^	0.78 ± 0.04^b^	0.91 ± 0.03^d^	1.97 ± 0.12^b^	0.32 ± 0.04^a^	0.39 ± 0.04^bc^	0.49 ± 0.03^d^
	1.0	0.6 ± 0.2^f^	0.9 ± 0.6^f^	4.1 ± 3.5^d^	0.12 ± 0.03^e^	0.80 ± 0.04^de^	1.09 ± 0.08^e^	0.12 ± 0.04^ab^	0.18 ± 0.03^e^	0.45 ± 0.01^e^
NEE^*^		1.07 ± 0.24^b^	1.30 ± 0.07^b^	8.78 ± 0.05^a^	0.28 ± 0.07^b^	0.52 ± 0.02^a^	0.52 ± 0.01^a^	0.27 ± 0.04^a^	0.28 ± 0.01^a^	0.29 ± 0.06^a^

* NEE, Non-encapsulated extract. The data represent the average value ± standard deviation of three replicates from each sample. The different letters within columns for primary or secondary liposomes and within row for the non-encapsulated extract represent significant differences between the means of different concentrations and stages of digestion using TPC, DPPH, and FRAP (*P* < 0.05).

Furthermore, secondary liposomes showed a significant increase in PSPE bioaccessibility regarding TPC and antioxidant activity as well; they protected more bioactive compounds against digestion than primary liposomes because the chitosan coated the liposome surface, which assisted in resisting the acidic enzymatic degradation of PSPE on the liposome surface in the gastric stage. Additionally, the bioaccessibility of PSPE for primary and secondary liposomes increased approximately 5 and 2 times, respectively. Moreover, the antioxidant activity (DPPH and FRAP assays) of PSPE increased almost twice in the primary and secondary liposomes. However, in secondary liposomes, the content of all phenolic compound concentrations was lower than that in PSPE and primary liposomes. Similar to our findings, those of Toro-Uribe et al., 2019, who investigated the bioaccessibility and antioxidant activity of free and liposomal forms of procyanidins, discovered that all liposome formulations displayed higher bioaccessibility and antioxidant activity in comparison to their respective counterparts in the non-encapsulated form under an *in vitro* digestion model (32). This can be explained by the interaction between phenolics and chitosan, which protects phenolic compound liposomal encapsulation from deleterious degradation agents (33). Additionally, the development of the polyphenol-chitosan complex limited the antioxidant activity. In addition, secondary liposomes had less TPC and lower antioxidant activity (DPPH and FRAP assays) than primary liposomes and non-encapsulated PSPE because the surface coating of liposomes with chitosan could prevent direct interaction of bile salts with the lipid membrane (34). The bioaccessibility of PSPE by TPC at all primary and secondary liposome concentrations was relevant only at 0.4 and 1% for primary and secondary liposomes, respectively. In contrast, bioaccessibility by DPPH and FRAP at all primary and secondary liposome concentrations was relevant and had a significant effect.

### Fourier transform infrared spectrometry (FTIR)

**Figure 1:**
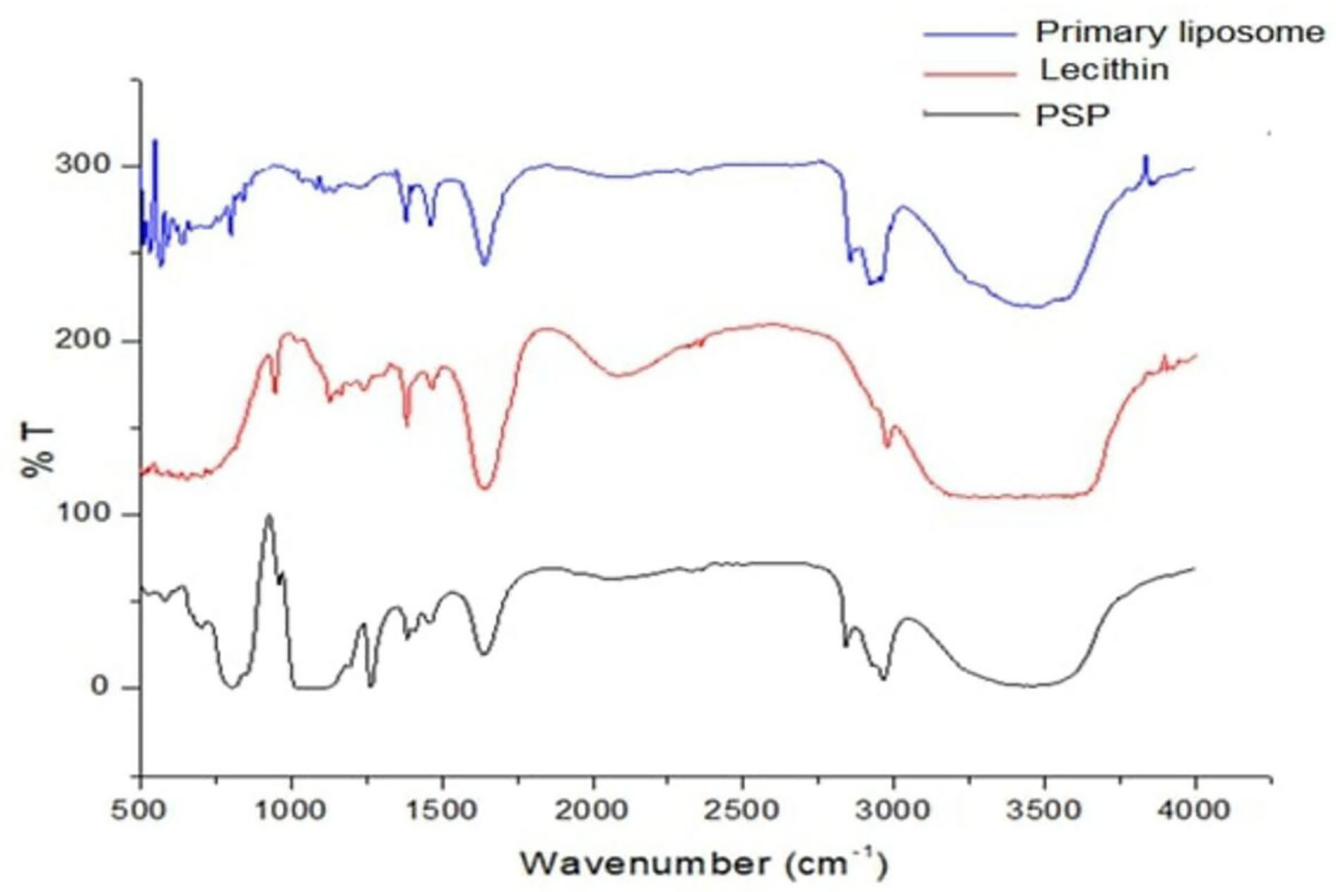
Fourier transform infrared spectrometry spectrum for PSPE/lecithin and primary liposomes. PSPE, Palm seed polyphenol extract; %T, %transmittance.

**Figure 2:**
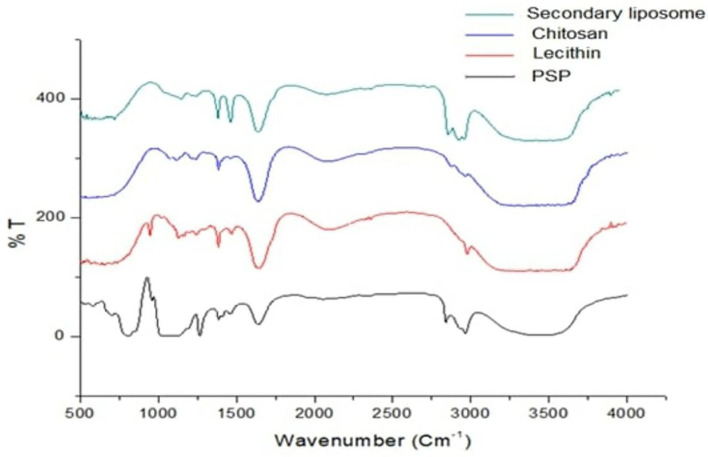
Fourier transform infrared spectrometry spectrum for chitosan/PSPE/lecithin and secondary liposomes.

The FT-IR spectra of PSPE/lecithin/primary liposome and PSPE/lecithin/chitosan/secondary liposome nanoparticles shown in Figures 1, 2, respectively, were investigated using their solid form to avoid being impacted by significant water absorption.

IR spectra were collected in the range 4,000–500 cm^−1^, but the bands in the range 4,000–1400 cm^−1^ were investigated in depth because they are typical of OH groups, while NH groups emerged in the range (3,000–4,000 cm^−1^) of different protonic species that undergo hydrogen bonding interactions. Another area of interest was the 1,800–1,400 cm^−1^ range, typical of the bending vibrations of the same group.

The most intense bands in the lecithin IR spectra refer to (i) the alkane bands correlating to the antisymmetric CH_2_ and CH_2_ scissoring vibrational modes at 2977.55 and 1467.56 cm^−1^, respectively; (ii) the carbonyl stretching vibration, located at 1635.34 cm^−1^; and (iii) the highly overlapped PO_2_ and P-O-C infrared active vibrations in the region between 1240 and 946 cm^−1^.

The FTIR spectra of chitosan membranes without ionic linking (Figure 2) showed broad bands at 3660.23 and 3297 cm^−1^ that were ascribed to O-H and N-H stretching vibrations of functional groups involved in hydrogen bonding. Discrete bands at 2965.98 and 2877.27 cm^−1^ were found and ascribed to C-H stretching vibrations. They also had distinct absorption bands at 1635.34 cm ^−1^ (C=O stretching in the amide group, amide I vibration). In other words, contributions from both species, protonated amino (-NH) groups and acetyl groups (R-C=O), demonstrate that chitosan is not completely deacetylated. The absorption of amide III vibration was 1384.64 cm^−1^ and and 1116.58 cm^−1^ absorption band (antisymmetric stretching of the C-O-C bridge owing to saccharide structure). Similarly, the signal at 894 cm^−1^, owing to the pyranose ring, showed the presence of the chitosan moiety (35).

The peak of the phenolic OH groups at 3430.74 cm^−1^ in the raw material vanished (36), and the peak at the absorbance region of 2964.05–2840.63 cm^−1^ showed the free vibrations of N-H stretching. The FTIR spectrum of PSPE also showed the classic amide bands, namely, amide I (1637.27 cm^−1^), amide II (1457.92 cm^−1^), and the bands at 1093.44 cm^−1^, which suggest an unsystematic coil shape.

Remarkable changes can be seen in the infrared absorption spectra due to the incorporation of PSE extract in lecithin (Figure 2); the broadband corresponding to OH groups is shifted from 3430.74 to 3486.67 cm^−1^, and the C=O bands at 1637.27 and 1641.13 cm^−1^ disappear. The band corresponding to C = O stretching in phospholipids shifted from 1650 to 1634 cm^−1^, the band at 1226.5 cm^−1^ shifted to 1225 cm^−1^, the band at 1232 cm^−1^ disappeared, and the band at 1093.44 cm^−1^ shifted to 1085.73 cm^−1^.

In the FTIR spectrum of the chitosan/PSPE nanoconjugate, the PSPE absorbance peak for N–H stretching at 2925.89 cm^−1^ was reduced and shifted to 3390.24 cm^−1^ (chitosan/PSPE). In addition, the amide I band in PSPE at 1637.27 cm^−1^ was also shifted to a lower wavenumber (1635.34 cm^−1^) in PSPE/chitosan (37, 38).

### Morphology

**Figure 3:**
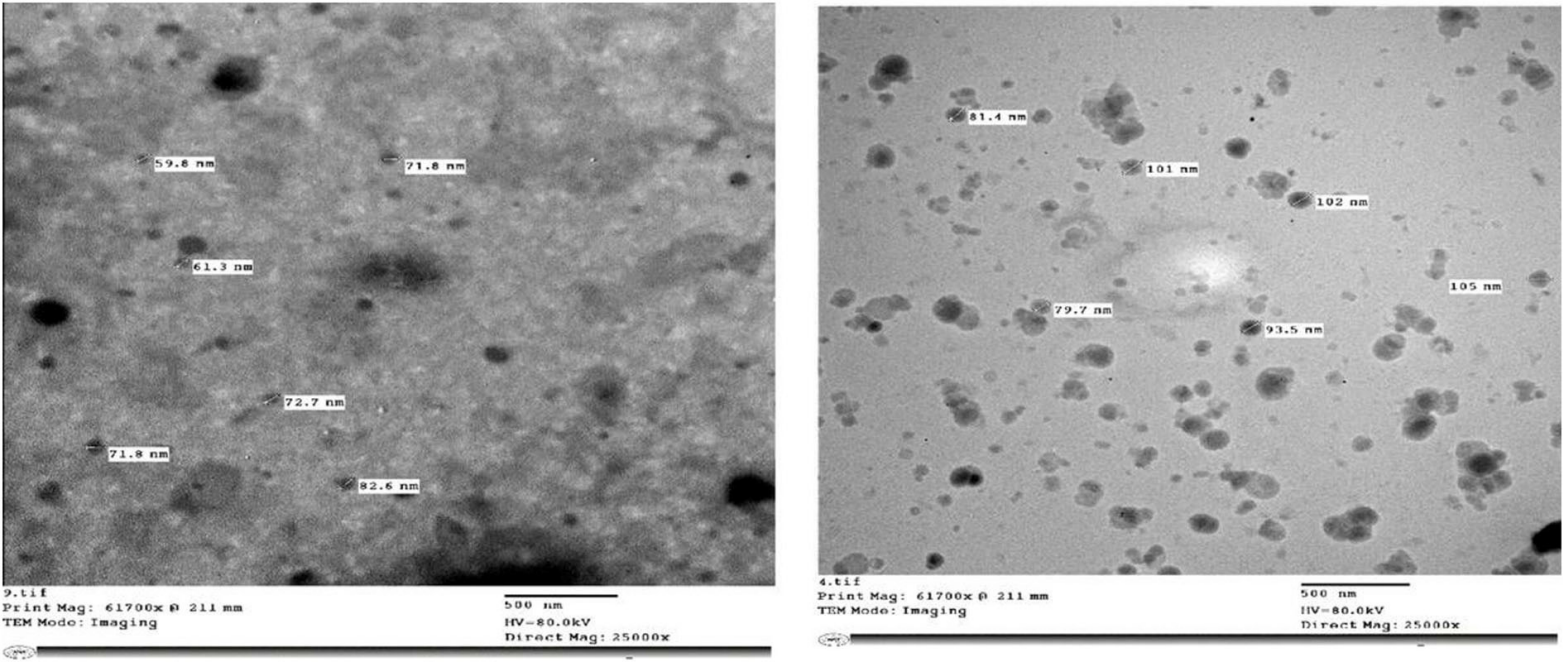
Transmission electron microscopy micrograph of PSPE liposomes with 0.2% was obtained at a scale of 500 nm, with a direct magnification of 25,000 ×.

Figure 3 illustrates the TEM image of PSPE-loaded liposomes containing 0.2% PSPE. The detected liposomes were circular, consistent with Thompson and Singh's results (39). In addition, the detected liposomes had mean sizes of approximately 80–100 nm for primary and secondary liposomes, respectively; these were smaller than the mean sizes for the same liposomes detected by laser scattering, 101–177 nm for primary and secondary liposomes, respectively.

### Yogurt's physical and chemical properties

Table 3 illustrates that the pH and the acidity% of the plain and yogurts fortified with secondary liposomes (high encapsulation efficiency) were analyzed over 14 days at 4°C. The pH values declined with increasing acidity% values during storage. The changes in the pH and acidity% are based on the time-dependent storage in which the yogurt starter inverts lactose to organic acids, mainly lactic acid, decreasing the pH-values. The present data showed that adding PSPE produces minimal changes in pH and acidity. These results agreed with those of Tavakoli et al. (40). They found that adding olive leaves (free and encapsulated) to yogurt also resulted in minimal variations in acidity and pH.

**Table 3:** The pH, acidity%, and syneresis of stirred yogurt fortified with PSPE-loaded secondary liposomes during storage.

**Parameter**	**Storage day**	**Control**	**T1**	**T2**	**T3**
pH	0	4.55 ± 0.02^c^	4.63 ± 0.02^b^	4.64 ± 0.01^b^	4.78 ± 0.02^a^
	7	4.50 ± 0.01^b^	4.63 ± 0.08^ab^	4.63 ± 0.06^ab^	4.77 ± 0.01^a^
	15	4.39 ± 0.01^c^	4.46 ± 0.01^b^	4.46 ± 0.01^b^	4.53 ± 0.01^a^
Acidity%	0	0.97 ± 0.04^a^	0.93 ± 0.04^a^	0.94 ± 0.04^a^	0.83 ± 0.04^b^
	7	0.98 ± 0.01^a^	0.96 ± 0.01a^b^	0.96 ± 0.01^ab^	0.89 ± 0.05^b^
	15	1.05 ± 0.06^a^	1.04 ± 0.03^a^	1.03 ± 0.08^a^	0.93 ± 0.03^a^
Syneresis%	0	29.6 ± 0.6^a^	24.8 ± 1.2^b^	21.3 ± 0.6^c^	20.4 ± 0.6^c^
	7	35.4 ± 0.6^a^	32.1 ± 0.6^b^	20.83 ± 0.01^c^	22.9 ± 0.6^c^
	15	36.7 ± 1.2^a^	33.8 ± 0.6^b^	25.4 ± 0.6^c^	27.5 ± 1.2^c^

The data represent the average value ± standard deviation of three replicates from each sample. Different letters in the rows represent statistically significant differences (*P* < 0.05). Control: yogurts without addition, T1, T2, and T3: yogurts containing 1.25, 2.5, and 3.75 g (PDSPE encapsulated with secondary liposomes, respectively).

### Yogurt syneresis

The syneresis rate of plain and fortified yogurts with secondary liposomes stored at 4°C is also reported in Table 3. The findings revealed that fortifying yogurt with secondary liposomes considerably reduced the rate of syneresis (*P* < 0.05). The T3 sample had the lowest syneresis readings and the most prolonged storage. This impact on the syneresis rate could be due to the increment of the total solids, enhancing yogurt consistency. Furthermore, the measured syneresis rate agrees with the results of Tavakoli et al. (40) in their investigation of the effect of nano-liposomes-encapsulated PSPE extracts on the quality of yogurts.

### Texture analysis

Table 4 shows the results of the textural analysis of plain and fortified yogurt with PSPE-loaded secondary liposomes. The hardness of the yogurt samples was reduced by increasing the concentration of PSPE liposomes. However, there were no significant differences (*P* < 0.05) in the hardness parameter of T1 containing 1.25 g PSPE-loaded secondary liposomes and the reference yogurt during storage.

**Table 4:** Texture analysis of yogurt supplemented with PSPE-secondary liposomes during storage.

**Samples**	**Hardness (*N*)**	**Cohesiveness (B/A area)**	**Springiness (Mm)**	**Gumminess (*N*)**	**Chewiness (N/m)**
**Fresh**				
C	1.00 ± 0.07^a^	0.89 ± 0.01^a^	0.62 ± 0.03^a^	0.71 ± 0.04^a^	0.43 ± 0.05^a^
T1	0.90 ± 0.07^a^	0.78 ± 0.00^b^	0.55 ± 0.01^b^	0.62 ± 0.01^b^	0.35 ± 0.03^b^
T2	0.70 ± 0.05^b^	0.70 ± 0.01^c^	0.54 ± 0.01^b^	0.49 ± 0.05^c^	0.28 ± 0.01^c^
T3	0.70 ± 0.14^b^	0.49 ± 0.01^d^	0.42 ± 0.02^c^	0.48 ± 0.02^c^	0.21 ± 0.01^d^
**15 days**				
C	0.80 ± 0.07^a^	0.89 ± 0.01^a^	0.84 ± 0.01^a^	0.63 ± 0.02^a^	0.51 ± 0.01^a^
T1	0.73 ± 0.17^b^	0.57 ± 0.02^b^	0.76 ± 0.01^b^	0.45 ± 0.03^b^	0.36 ± 0.00^b^
T2	0.72 ± 0.03^b^	0.38 ± 0.02^c^	0.55 ± 0.02^c^	0.26 ± 0.02^c^	0.34 ± 0.01^b^
T3	0.62 ± 0.04^c^	0.20 ± 0.04^d^	0.51 ± 0.01^d^	0.14 ± 0.00^d^	0.14 ± 0.01^c^

The data represent the average value ± standard deviation of three replicates from each sample. The different letters in the columns represent statistically significant differences (*P* < 0.05).

On the other hand, hardness significantly decreased (*P* < 0.05) between yogurts samples T1, T2, and T3 containing 1.25, 2.5, and 3.75 g PSPE-loaded secondary liposomes, respectively, compared to the reference. This decrease is due to the weakness of the protein network because it does not easily interact with casein in the yogurt matrix, as it is protected by the encapsulation material, as indicated in (41). The variance in the other textural parameters had the same trend as hardness. Thus, the cohesiveness, springiness, gumminess, and chewiness values were reduced with the increased amount of PSPE-loaded secondary liposomes. All values were reduced in each yogurt sample during storage, but the changes were more observable in plain yogurt. Mean cohesiveness values were more reduced for yogurt samples fortified with PSPE-loaded secondary liposomes than for plain yogurt; this may be due to the reduced force of protein-protein bonds (42). Springiness showed the same trend. The chewiness and gumminess values of yogurt fortified with PSPE-loaded secondary liposomes significantly differed from those of plain yogurt. Overall, yogurt fortification with lyophilized PSPE reduced the texture profile of the yogurt compared with the reference, both freshly produced and after storage. These results agree with El**-**Said et al. (27): adding 5% Doum extract liposomes in yogurt manufacture led to a slight effect on the development of the textural profile of yogurt compared to the control. This may be attributed to the moisture content of fresh samples having a higher concentration, which weakens the protein network, resulting in a lower firmness.

### Color parameters

Food appearance is crucial to customer acceptability; the color of yogurt is one of the most significant quality factors. Table 5 shows the color characteristics of yogurts (L^*^ [lightness], a^*^ [red/greenness], and b^*^ [yellow/blueness]). In this research, plain yogurt (C) had the greatest L^*^ values, indicating that it has the brightest color, followed by the T1, T2, and T3 yogurt samples. Thus, the results show that the addition of secondary liposomes reduced brightness (L^*^ values). Furthermore, yogurt samples fortified with PSPE-loaded secondary liposomes reduced the negative a^*^ and the positive b^*^ values comparable to plain yogurt.

**Table 5:** Change in color parameters of stirred yogurt fortified with PSPE-loaded secondary liposomes.

**Samples**	**Color parameters**		
	L*	a*	b*
Control	83.70 ± 0.11^a^	−2.94 ± 0.02^d^	9.99 ± 0.01^a^
T1	82.18 ± 0.05^b^	−2.20 ± 0.02^c^	9.38 ± 0.01^b^
T2	80.21 ± 0.11^c^	−1.46 ± 0.02^b^	8.31 ± 0.02^c^
T3	79.89 ± 0.03^d^	−1.21 ± 0.01^a^	8.00 ± 0.02^d^

The data represent the average value ± standard deviation of three replicates from each sample. The different letters in the columns represent statistically significant differences (*P* < 0.05).

Some previous findings demonstrated that adding plant extracts modified the color properties of yogurt (43). Using Aronia juice, Nguyen and Hwang (44) also obtained yogurt with a deeper, more intense red color than the reference yogurt. Encapsulation techniques have recently been employed to prevent color changes and bitterness caused by polyphenol extract added to dairy products. In this investigation, the secondary liposomes efficiently disguised the brown color of PSPE and, consequently, prevented color changes in yogurt. Tavakoli et al. (40) reported a purer whiteness in plain yogurt than in yogurt enriched with free olive leaf phenolics and nano-liposomes. Furthermore, adding the non-encapsulated PSPE extract significantly reduced the L^*^ values, whereas adding nano-liposomes only marginally reduced them.

### Yogurt sensory analysis

**Figure 4:**
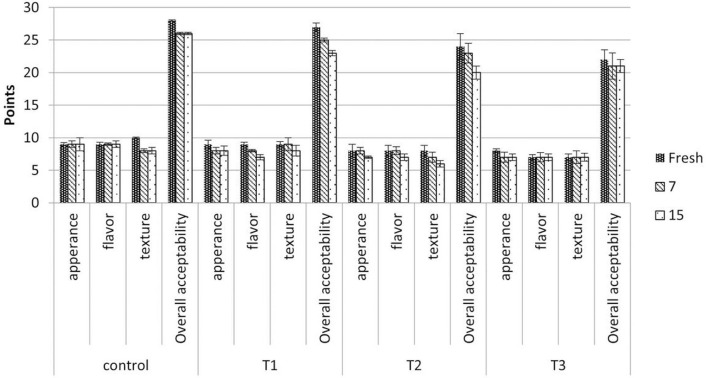
Effect of PSPE-loaded secondary liposomes on the sensory characteristics of stirred yogurt samples. Control: Plain yogurt without liposomes, T1, T2, and T3, yogurts containing 1.25, 2.5, and 3.75 g (lyophilized secondary liposome) equivalent to 25, 50, and 75 mg of phenolic compounds, respectively. The data represent the average value ± standard deviation of three replicates from each sample.

Ten panelists assessed the sensory qualities of all manufactured yogurts. The scores of the sensory attribute criteria were evaluated, and the findings are displayed in Figure 4. According to the results, there were significant alterations in aroma and flavor. All yogurt samples enriched with PSPE-loaded liposomes, except for the reference yogurt, varied in appearance, flavor, and texture. According to the results, adding PSPE-loaded secondary liposomes to yogurt did not affect the texture as it did the appearance and flavor. All panelists agreed that the control and T1 samples were the best in quality, with T3 obtaining the lowest score. According to Ghorbanzade et al. (45), nano-encapsulation of fish oil with nanoliposomes did not affect the general acceptability of yogurt samples following storage.

## Conclusion

This study investigated the potential application of date seeds as a source of polyphenols and bioactive compounds. The effect of ultrasound-assisted extraction and encapsulation by the liposome technique was described. The palm seed phenolic extraction was successfully protected by microencapsulation with liposomes, which was proven by *in vitro* digestion and FTIR analysis. However, to achieve higher protection of bioactive compounds in the encapsulation system during storage as well as an advantageous release profile for *in vitro* digestion, liposomes have to be coated with chitosan to form two-layer liposomes. Phenolics extracted and lyophilized from date seed powder and preserved by liposomes could be used due to their health benefits and antioxidant activity in the gut phase. Furthermore, encapsulating palm seed phenolic extraction using the liposome technique effectively generates an innovative functional food of yogurt with improved quality characteristics and valuable functions.

## Data availability statement

The raw data supporting the conclusions of this article will be made available by the authors, without undue reservation.

## Author contributions

MH, TE-M, DB, and LN contributed to the conception and design of the study. MB performed the statistical analysis. MH wrote the first draft of the manuscript. TE-M, XH, and MB wrote sections of the manuscript. All authors contributed to manuscript revision, read, and approved the submitted version.

## Conflict of interest

The authors declare that the research was conducted in the absence of any commercial or financial relationships that could be construed as a potential conflict of interest.

## Publisher's note

All claims expressed in this article are solely those of the authors and do not necessarily represent those of their affiliated organizations, or those of the publisher, the editors and the reviewers. Any product that may be evaluated in this article, or claim that may be made by its manufacturer, is not guaranteed or endorsed by the publisher.
